# The Role of Neuropeptides in Suicidal Behavior: A Systematic Review

**DOI:** 10.1155/2013/687575

**Published:** 2013-08-06

**Authors:** Gianluca Serafini, Maurizio Pompili, Daniel Lindqvist, Yogesh Dwivedi, Paolo Girardi

**Affiliations:** ^1^Department of Neurosciences, Mental Health and Sensory Organs, Suicide Prevention Center, Sant'Andrea Hospital, Sapienza University of Rome, 1035-1039 Via di Grottarossa, 00189 Rome, Italy; ^2^Department of Clinical Sciences, Section of Psychiatry, Lund University, SE-221 85 Lund, Sweden; ^3^Psychiatric Institute, Department of Psychiatry, College of Medicine, University of Illinois at Chicago, Chicago, IL 60612, USA

## Abstract

There is a growing evidence that neuropeptides may be involved in the pathophysiology of suicidal behavior. A critical review of the literature was conducted to investigate the association between neuropeptides and suicidal behavior. Only articles from peer-reviewed journals were selected for the inclusion in the present review. Twenty-six articles were assessed for eligibility but only 22 studies were included. Most studies have documented an association between suicidality and some neuropeptides such as corticotropin-releasing factor (CRF), VGF, cholecystokinin, substance P, and neuropeptide Y (NPY), which have been demonstrated to act as key neuromodulators of emotional processing. Significant differences in neuropeptides levels have been found in those who have attempted or completed suicide compared with healthy controls or those dying from other causes. Despite cross-sectional associations between neuropeptides levels and suicidal behavior, causality may not be inferred. The implications of the mentioned studies were discussed in this review paper.

## 1. Introduction

Suicide represents one of the leading causes of premature death in the general population. Higher suicide rates have generally been reported in men compared to women in almost all countries, with elderly men at especially high risk for completed suicide [1–3]. Rates of attempted suicide average 20–30 times higher than rates of completed suicide in the general population but are probably underreported and range widely in method and seriousness of intent [4, 5]. Most cases of suicide involve psychiatric conditions, nearly half of which (48.5%) suffered from mood disorders [6].

Major depressive disorder (MDD) is a chronic and invalidating disease associated with significant functional impairment and occupational disability. Individuals with MDD are at increased risk for suicidal behavior [7–13]. Impulsivity and loss of impulse control may be considered important dimensions of suicidality. As opposed to psychiatric disorders such as MDD that may be regarded as more proximal risk factors for suicide, some have suggested that impulsivity may be seen as a personality characteristic and thus a more distal risk factor for suicide [14]. Yet others have emphasized the importance to distinguish between attempt impulsivity (state) and attempter impulsivity (trait) in understanding suicide attempts [15]. Impulsive personality characteristics have been associated with a higher likelihood of suicidal behavior across nosological entities and also in nonpsychiatric populations [16]. The structure of impulsivity in severe depression as well as its relationships to suicide attempts have been investigated by Corruble et al. [17]. They found that recent suicide attempts in severely depressed individuals were related to loss of control and cogntive impulsivity but not to nonplanning. The tendency to engage in more impulsive behaviors, reflecting possible loss of impulse control, may be assessed using the impulse control scale [18]. Behavioral loss of control has been found as a state-dependent dimension of impulsivity and suicide attempts were mainly related to loss of control in depressed patients. The planning subscale of the suicide intent scale [19] can be used to objectify degree of impulsivity during a suicide attempt.

Despite the recent advancements in the understanding of suicide, our current knowledge concerning the neurobiology of this complex phenomenon is still limited.

Peptide hormones might play a critical role in normal as well as pathological functions of the central nervous system [20, 21] acting as selective messengers in specific emotive circuits of the brain [22] and function as crucial mediators of the stress response and adaptation [23]. Neuropeptides show high specificity for their target receptors and are highly potent; also, they are not accumulated in tissues as they are efficiently metabolized by endogenous enzymes [20]. They generally interact with metabotropic or G-protein-coupled receptors, whereas neurotransmitters are chemical substances that affect the excitability of neurons acting as mediators of the neurotransmission through synapses. Neuropeptides are generally stored in large dense-core vesicles; conversely, neurotransmitters are secreted in small synaptic vesicles mainly located in clusters at presynaptic locations. It has been suggested that peptide signals play a prominent role in the information processing, that is, quite unlike that of conventional neurotransmitters [24].

Neuropeptides may modulate a more prolonged transmission than typical neurotransmitters showing relevant neurotrophic effects [25]. Neuropeptides are mainly associated with one or more neurotransmitters which serve to integrate their modulatory functions. The high frequency release of neuropeptides is in line with the ability to mediate adaptation to homeostatic challenges [23]. Some authors suggested that cholecystokinin (CCK) and neuropeptide Y (NPY) are promising options for the discovery of intriguing and more selective pharmacological options [26]. Other neuropeptides like corticotropin-releasing factor (CRF) and neurokinin 1 (NK1) substance P systems are known to modulate 5HT neurons through actions within the dorsal raphe (DR) networks [27]. A hyperactivity of central neuropeptidergic circuits such as CRF and arginine vasopressin (AVP) systems may play a key role in major affective and anxiety disorders [25, 28, 29]. These neuropeptidergic systems may be involved in the action of antidepressant drugs, as demonstrated by the paroxetine-induced reduction of vasopressinergic overexpression [30]. Neuropeptides dysregulation may be also involved in suicidal behavior. Sherrin et al. [31] compared CCKB receptor gene expression in ten suicide victims compared with ten matched controls using quantitative PCR and showed increased gene expression in suicide victims in prefrontal and cingulate cortex as well as cerebellum. This study was in line with previous results demonstrating increased CCK receptor binding in frontal and cingulated cortex of suicide victims [32]. Also, recent suicide attempts have been associated with lower plasma levels of NPY [33]. Lower NPY levels have been found in prefrontal cortex and caudate nucleus of suicide victims compared to age-matched subjects with other causes of death [34]. Reduced NPY levels, which are widely distributed throughout the central nervous system, have been found in depressed patients compared to controls [35], as well as in the brain of suicide victims compared to those who died a natural or accidental death [34].

To what extent neuropeptide abnormalities may be considered reliable predictors of suicidal behavior is, however, a matter of debate. Considering this background, we aimed to critically review the current literature about the role of neuropeptides in suicidal behavior.

## 2. Methods

### 2.1. Information Sources, Search Strategy, and Study Selection

**Figure 1:**
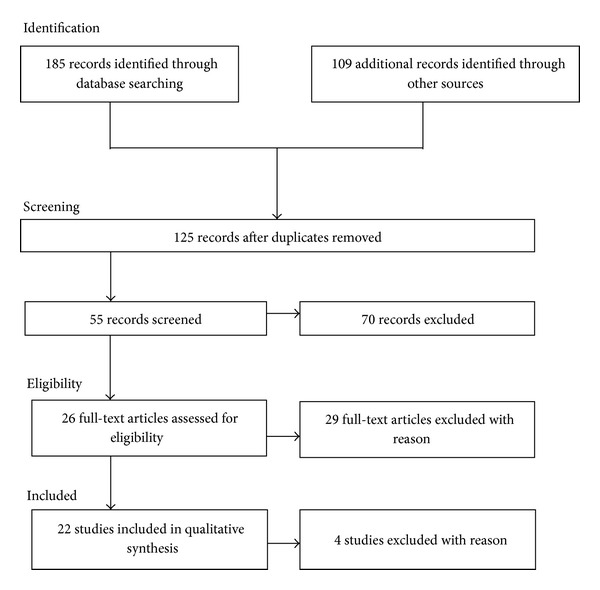
Flowchart of the search and selection process identification.

A detailed search strategy summarized in Figure 1 was used to identify relevant studies. The possible involvement of neuropeptides in suicidal behavior was investigated using a detailed PubMed/MEDLINE, Scopus, PsycLit, and PsycInfo search to identify all papers and book chapters in English language during the period between January 1985 and January 2013. The search used a combination of the following terms: “Neuropeptides” OR “Central nervous System Peptides” OR “Small bioactive peptides” AND “Suicid*” (including suicidal behaviors OR suicide ideation OR suicidal thoughts OR deliberate self-harm OR suicidal attempts). Two independent researchers conducted a two-step literature search. Any discrepancies between the two reviewers who, blind to each other, examined the studies for the possible inclusion were resolved by consultations with a senior author. The reference lists of the articles included in the review were also manually checked for relevant studies.

Studies were included according to the following criteria: (a) being an original paper in a peer-reviewed journal and (b) have analyzed the possible involvement of neuropeptides in suicidal behavior. Figure 1 summarized the search strategy used for selecting studies (identification, screening, eligibility, and inclusion process) in the review.

### 2.2. Study Design and Eligibility Criteria

To achieve a high standard of reporting, we have adopted Preferred Reporting Items for Systematic Reviews and Meta-Analyses, (PRISMA) guidelines [36]. The PRISMA Statement consists of a 27-item checklist and a four-phase flow diagram for reporting in systematic reviews and meta-analyses. PRISMA includes the broader effort to improve the reporting of different types of health research and in turn to improve the quality of research used in decision making in healthcare.

### 2.3. Recorded Variables

The recorded variables for each article about neuropeptides and suicide were sample characteristics, study design, type of neuropeptide which has been investigated, main findings, limitations, and conclusions (Table 2).

## 3. Results

### 3.1. Number of Studies Selected

The combined search strategies yielded a total of 185 articles of which, after a complete analysis, 26 full-text articles were screened and considered for the inclusion in the current review. 159 studies were excluded because they were considered not relevant to the main topic. Specifically, we excluded articles not published in peer reviewed journals and not in English language, articles without abstracts, abstracts that did not explicitly mention the link between neuropeptides and suicide, articles with a publication date before 1985, and those with unclear data regarding materials and methods and number of patients analyzed. As mentioned, we assessed 26 articles for eligibility but 4 full-text articles were excluded due to low relevance to the main theme leaving 22 studies that fulfilled our inclusion criteria. 

### 3.2. Corticotropin-Releasing Factor (CRF), Oxytocin, and Arginine Vasopressin (AVP)

CRF, oxytocin, and AVP have been hypothesized to play a crucial role in the pathogenesis of major affective disorders and presumably suicidal behavior.

Clinical and basic research supports the notion that these neuropeptides appear crucial in modulating behavior. During the past decade, drug discovery efforts using neuropeptide receptor ligands have focused on a limited number of neuropeptides, such as CRF, oxytocin, and AVP although many other neuropeptide systems have been identified.  Table 1 summarized the large body of evidence suggesting the involvement of CRF, oxytocin, and AVP in major affective disorders and suicidal behavior.

### 3.3. Galanin

Galanin (GAL) is a 30-amino-acid peptide that binds GAL receptor1 (R1), GALR2, and GALR3 found along the HPA axis and involved in several human functions such as food and alcohol intake, metabolism, osmoregulation, seizure threshold, and reproduction. Depression-like behaviors seem to be induced by the activation of GALR1 and attenuated depression-like behaviors derived by the activation of GALR3 [103]. GAL immunoreactivity has been showed in human midbrain and limbic areas [104]. Although several studies sustained a role of GAL in the hypothalamic-pituitary-adrenal (HPA) axis activity, findings about this association are quite conflicting [105]. GAL has been reported to modulate serotonergic and noradrenergic circuits [106] as well as GAL transmission (related to the inhibition of dopaminergic neurons in the ventral tegmental area) mediating anhedonia and reduced locomotor activity in depressed patients enhanced locus coeruleus (LC) hyperactivity [107]. GAL antagonists have been reported to improve these effects [107]. The expression of somatodendritic 5HT1a autoreceptors (significantly involved in the pathophysiology of major depression) in the DR nucleus may be modified by GAL with a consequent enhancement of serotonergic transmission [108]. Anxiolytic and antidepressant properties have been reported using GALR3 antagonists in animal models [109]. Finally, evidence suggested that activation of GALR2 is associated with neurogenesis and neuroprotective effects improving hippocampal damage which was commonly found after chronic depression [110]. 

### 3.4. Substance P

SP that binds three different g-protein-coupled tachykinin receptors—NK1, NK2, and NK3, has been associated with nociception, respiration, cardiovascular and thermoregulation, gut motility, emetic response, and stress-related disorders [111]. The SP/NK1 system modulates central noradrenergic and serotonergic circuits. Serotonergic activity in the hippocampus and lateral septum was enhanced by NK1 antagonism [112]. NK1 immunoreactivity has been found in the noradrenergic neurons of LC [113]. Several studies sustained a role of SP/NK1 system in the HPA axis and stress responsiveness but not all studies confirmed these findings [111]. Stressful situations may stimulate SP/NK1 system and enhance SP release in the hypothalamus, amygdala, lateral septum, nucleus accumbens, and LC [114]. Animal models have shown that NK1 antagonist may reduce anxiogenic effects related to the injection of SP in the central nucleus of the amygdala [115] showing antidepressant properties [116]. Furmark et al. [117] using functional imaging also suggested that NK1 antagonism in humans reduced social anxiety related to the modulation of serotonergic circuits. NK1 antagonists represent promising medications with consistent antidepressant and anxiolytic properties. There are, however, conflicting results based on the main studies [114, 115] measuring SP levels among psychiatric patients and controls. Animal models of anxiety and depression have shown preclinical efficacy by NK2 antagonists [118] whereas NK3 receptors might be valid targets for antipsychotic treatment [119]. 

### 3.5. Neuropeptide Y

NPY is a 36-amino-acid which is involved in circadian rhythms, neurogenesis and neuroprotection, nociception, feeding behavior and energy regulation, neuronal excitability, emotion and cognition, stress response, and resilience. NPY action is associated with several neurotransmitters such as GABA, serotonin, norepinephrine, and other catecholamines [120, 121]. The central NPY transmission has been associated with anxiolytic and antidepressant properties in animal models [122]. Anxiolytic and antidepressant effects have been demonstrated after the activation of receptors Y1 in animals [123] whereas the activation of Y2 is related to anxiogenic effects [124]. Recently, conflicting results have emerged after activation or Y5 antagonism [125]. 

The enhanced NPY transmission might be stimulated to control stress response through the modulation of CRF and noradrenergic tone. In fact, the reduced NPY transmission may be related to increased CRF and noradrenergic transmission determining anxiety and depression behaviors [126]. NPY, no doubt, has important and complex interactions with the CRF system. Sajdyk et al. [127] suggested that NPY is associated with anxiolytic activity in the amygdala where both CRF receptors and high concentrations of NPY have been found. However, mixed findings regarding NPY levels have been reported among humans [128]. 

### 3.6. Cholecystokinin

CCK is a 33-amino-acid peptide that has been found to be implicated in gastric emptying, gallbladder contraction, pancreatic enzyme release, and suppression of appetite. CCK levels were observed in the cortex, hippocampus, amygdala, nucleus accumbens, striatum, and substantia nigra [129]. CCKR1 and CCKR2, with the latter predominantly expressed in the brain, have been described as the 2 G-protein coupled receptors binding CCK. Relevant effects on behaviors which are known to be mediated via dopamine seem to be induced by CCK peptides.

The interaction of CCK with CCKR2 has been reported to inhibit dopamine release blocking dopamine-mediated behaviors into the anterior nucleus accumbens whereas the binding with CCKR1 into the posterior nucleus accumbens is associated with opposite effects [130, 131]. CCK agonists that may be active in the treatment of schizophrenia should be nonselective or CCKR1 selective. Overall, there are evidence suggesting that CCK may be involved in both affective- and stress-related disorders. 

### 3.7. Dynorphins

The complete sequence of dynorphin A (Dyn-A) (17amino acids) was identified by Goldstein et al. [132] in 1981. The prefix dyn—was taken from the Greek (power) whereas—orphin suggests its opioid nature. Dyn-A might play a regulatory role in epilepsy and addiction, whereas findings related to emotional control are quite conflicting. Data are mainly derived from animal models and a correct interpretation of findings from mouse testing has to be observed in the general context of a different social behavior in rats and mice [133]. Exploring the effects and molecular mechanism of action of dynorphins in humans is a very complicated task considering mood and anxiety behaviors that may be investigated only indirectly through a detailed interpretation of animal models.

### 3.8. Orexin

Orexins or hypocretins and their receptors have been discovered in 1998 as predominantly expressed in the lateral hypothalamus [134], specifically in the perifornical region of the lateral hypothalamus and the posterior hypothalamic area [135, 136]. The name hypocretins is related to the similar amino acid sequence of the gut peptide secretin, whereas the name orexins is derived by the Greek word orexis which means appetite (these are peptides that stimulate food intake) [137]. 

Hypocretin fibers such as the central nucleus of the amygdala (CeA), bed nucleus of the stria terminalis (BNST), LC, and paraventricular nucleus of the hypothalamus (PVN) might have a specific role in the regulationof stress responses [136]. Hypocretins bind the hypocretin-1 and hypocretin-2 receptors. Hypocretin-1 has a 2-3-fold higher affinity for the hypocretin-1 receptor than hypocretin-2 [137]. Several studies have provided evidence for a role of orexins/hypocretins in the regulation of stress responses and in the regulation of many human behaviors. 

### 3.9. The Association between Neuropeptides and Suicidality

Neuropeptides may play a key role in the stress response as well as in the regulation of several human behaviors. Table 2 summarized the most relevant studies addressing the association between neuropeptides and suicide. 

Important insight into the role of neuropeptides in the pathogenesis of MDD and suicidal behavior has been given. Orexin alterations have been investigated in several subgroups of patients. In a factor analysis of CSF biomarkers in suicide attempters, a negative loading on orexin, along with positive loading on proinflammatory cytokine interleukin-6 and monoamine metabolites 5-HIAA and HVA was associated with violent suicide method and risk for future suicide completion [83]. Orexin levels were also reported to be significantly lower in patients with MDD compared to those with adjustment disorder and dysthymia [87]. The same research group has also found that lower orexin levels were associated with depressive symptoms such as higher inertia and decreased motor activity in 101 suicide attempters [88]. As later reported by Brundin et al. [85], a significant negative correlation was found between the change in CSF-orexin levels and symptoms of suicidality, assessed by SUAS [138], one year after a suicide attempt. 

Furthermore, Inder et al. [93] found a significant positive correlation between plasma AVP, ACTH, and cortisol levels in depressed subjects. The authors reported that plasma AVP levels were increased in those subjects who had attempted suicide whereas no difference was observed in mean cortisol between depressed subjects and controls. 

Patients with NSSI were also investigated in order to find abnormalities in neuropeptides. Stanley et al. [84] recruited 29 psychiatric patients with a history of one or more suicide attempts with a mean of three attempts. Patients with NSSI had lower CSF *β*-endorphin levels and met-enkephalin when compared to a diagnostically matched group of patients without NSSI but no difference in dynorphin levels was found between the two groups. 

The excessive CRH secretion and neurotransmission which is involved in the pathophysiology of depressive disorders has been suggested to extend beyond the hypothalamus and to involve several extrahypothalamic brain regions. Austin et al. [89] found a significant positive correlation between the magnitude of the increase in CRH-IR levels in the LC and the age at illness onset in a sample of 11 depressed subjects who died by suicide compared with controls. Specifically, the mean level of CRH-IR in depressed subjects was significantly increased by 30% in the LC, by 45% in the caudal nucleus of the DR and by 39% in the median raphe relative to healthy controls. 

In addition, Träskman-Bendz et al. [100] reported that, in a sample of 44 suicide attempters, patients who had made previous suicide attempts had significantly lower CRH than those who had not, and only patients with MDD had significantly lower somatostatin, CRH, and DSIP compared to patients with other diagnoses. Westrin et al. [95] found that levels of CSF-somatostatin, but not CSF-CRH, increased with clinical improvement in suicidal patients independently of psychiatric diagnoses.

Westrin et al. [96] found that in a sample of 34 MDD suicidal patients, those with previous suicide attempts had increased plasma DSIP levels. Scarone et al. [101] suggested reduced beta-endorphin levels in the left temporal cortex, frontal cortex, and caudate nucleus in 7 suicides compared to 7 controls. An asymmetrical concentration of beta-endorphins in suicides (left less than right) was also suggested in frontal cortex and caudate nucleus.

Whether psychoactive treatments affect neuropeptides levels is a matter of debate. Olsson et al. [94] found that antidepressant treatment seemed to affect the levels of CSF NPY and SP. Westrin et al. [33] suggested that there may be alterations in CRH and NPY plasma levels in patients with a mood disorder who had recently attempted suicide. Specifically, the authors found that cortisol was significantly high and CRH and NPY were low in recent suicide attempters. Moreover, Westrin et al. [97] reported that NPY correlated positively with psychasthenia, irritability, and stability in patients and negatively with muscular tension, psychasthenia, verbal aggression, and irritability in controls. Cortisol correlated positively with validity, extraversion, and verbal aggression and negatively with inhibition of aggression in controls.

Also, post-mortem studies have been conducted in order to analyze the possible role of neuropeptides in suicidal behavior. Merali et al. [90] suggested that in a sample of 30 suicides and 37 controls, suicides were associated with site-specific alterations in the endogenous levels of CRH, AVP, GRP, and NMB. However, GRP and NMB variations were limited although GRP-ir within the LC of suicides was higher than in control subjects and NMB-ir which was reduced at the DVC of suicides. In another post-mortem study, Caberlotto and Hurd [92] reported that no significant alterations in Y1 or Y2 mRNA expression levels were observed between suicides and controls. The Y2 mRNA expression was increased in layer IV of suicides. There was a negative correlation between Y1 mRNA expression levels in the prefrontal cortex and increasing age.

Moreover, Widdowson et al. [34] reported the existence of NPY deficit in the brain leading to region-specific reductions in neuropeptide concentrations in individuals with a history of depression. In this post-mortem study, the authors found that NPY levels were significantly lower in the frontal cortex and caudate nucleus of suicide victims compared to age-matched controls. 

Negative findings have also been reported. Pitchot et al. [86] found no correlation between severity of depressive symptoms and AVP-neurophysins or post-DST cortisol levels in a sample of 28 MDD inpatients. Any correlation between AVP-neurophysins and post-DST cortisol levels (*r* = −0.07, *P* = 0.72) was observed. AVP neurophysins did not differ between DST suppressors and nonsuppressors. In addition, Brunner et al. [91] found no differences in CSF AVP concentrations between 19 depressed suicide attempters, depressed nonsuicidal patients, and 9 neurological control subjects and there was no relationship between CSF AVP and monoamine metabolites in CSF.

In a 5-year follow-up study, Roy [98] did not find evidence that NPY, somatostatin, diazepam-binding inhibitor, GABA, or CRH were major determinants of suicidal behavior or its repetition in depression, since no significant differences between depressed patients who did or did not reattempt suicide were found. Also, Ordway et al. [99] found no significant differences in NPY concentrations between suicide victims with MDD (with comorbid alcohol dependence) and age-matched control subjects. Finally, Maes et al. [102] reported no significant differences in TSH, FT4, ACTH, and total L-TRP levels between patients with suicidal ideation and those without.

## 4. Discussion

The present review supports a role of neuropeptides in the pathophysiology of suicidal behavior. Based on the included studies, there may be associations between neuropeptides and suicidal behavior but this does not imply the existence of a causal link. Most studies (thirtheen) [33, 34, 83–85, 89, 90, 92–96, 101] included in our review found an association between neuropeptides and suicidal behavior; however, there are also six studies [86, 91, 98–100, 102] that did not confirm this association. Moreover, there are other studies suggesting that neuropeptides abnormalities may be found in subjects with MDD but not suicidal behavior [87, 88, 97]. 

One relevant limitation of the selected studies is related to the variable neuropeptide concentrations (e.g., some studies reporting increases and others reporting reductions or no significant changes) in those who attempted suicide or died by suicide compared with healthy controls or those dying a sudden natural or accidental death, respectively. These findings reflected the significant heterogeneity in terms of psychopathology which is commonly observed in those with suicidal behavior together with the variability in the antidepressant response which may be found in these different subgroups of patients. Specific stressors may induce specific neuropeptide responses: SP may be evoked by structural damage, oxytocin release by social threats, melanin-concentrating hormone (MCH) by abnormal energy homeostasis, and AVP changes by blood volume changes, respectively. Neuropeptides such as CRF, VGF, CCK, SP, and NPY have been demonstrated to act as key neuromodulators of emotional processing [139, 140].

Neuropeptides may be considered as crucial molecules in the interaction between genes and environment as well as fundamental mediators of the stress response able to in turn affect gene regulation in order to respond to the different environmental stimuli. What is the main mechanism underlying neuropeptides alterations in subjects with suicidal behavior? Evidence suggests that modulation of monoaminergic transmission may represent the main mechanism by which the neuropeptide galanin was implicated in stress-related disorders [103]. This modulation of monoaminergic systems has been observed predominantly in the LC and DR nuclei. At the molecular level, stressful events may enhance the expression of tyrosine hydroxylase [141–143], the enzyme which is involved in controlling catecholamine synthesis, and GAL [103, 144] with the final increase of noradrenergic transmission. This may determine both direct and indirect inhibitory effects on postsynaptic neuronal functions implicated in depression-like behaviors.

The role of NPY and its receptors in affective disorders and stress-related conditions has been investigated for over two decades but still remains rather controversial. According to behavioral findings on NPY, Y1, and Y2 receptors in animal models, evidence suggested an important role of NPY in emotional responses and stress- or depression-related disorders [145]. Westrin et al. [33] reported significantly higher cortisol, CRH, and lower NPY levels in recent suicide attempters when compared with healthy controls. NPY was also reported by the same research group to correlate positively with psychasthenia, irritability, and stability and negatively with validity in patients [97]. There are also studies that did not report significant changes in neuropeptides concentrations in patients with suicidal behavior. There were no significant differences in CSF NPY, somatostatin, diazepam-binding inhibitor, GABA, or CRH levels between depressed patients who did or did not reattempt suicide during the follow-up or those who had never attempted according to Roy [98]. However, a nonsuicidal control group was not included in the mentioned study. Moreover, Ordway et al. [99] did not find an association between NPY and completed suicide, although the fact that some suicide victims had a history of comorbid alcohol dependency who may have confounded the results.

Pitchot and colleagues [86] found no association between major depressive symptoms as assessed by HDRS and AVP-neurophysins levels. However, the authors of this study stated that plasma AVP levels presumably did not reflect AVP activity in the mangocellular portion of the paraventricular nucleus. Also, no significant difference in dynorphin levels was found between patients with a history of one or more suicide attempts [84]. Here, it is important to note that plasma neuropeptide levels may not accurately reflect central neuropeptide levels. In addition, Westrin et al. [95] found no significant changes in CSF-CRH at follow-up in 16 patients of which eleven had made previous suicide attempts. However, the substances which have been ingested by patients may have caused the initially low CRH and somatostatin levels, and thus confounded the results. Furthermore, this study did not demonstrate whether somatostatin changes are specifically related to either suicidality or depression but only that somatostatin changes are related to clinical symptomatic recovery in suicidal patients.

Finally, based on Maes et al. investigation [102], no relevant (significant) differences were observed in terms of TSH, FT4, pre- and postdexamethasone cortisol, ACTH, and L-TRP levels between patients with suicidal ideation and those without. However, considering the possible caveats, this study was cross-sectional in nature and subjects were not assessed using psychometric instruments.

Many of the mentioned studies should be considered in the light of the following shortcomings. Many studies were not completely informative over time and lacked adequate long-term follow-up periods. The naturalistic designs may not completely control for the effect of confounding factors such as the duration of treatment and disease and the diagnostic subtypes. Also, samples were often too small or included mixed, heterogeneous patients and usually did not allow to generalize findings. Patients with suicidal behavior who were included in these studies may be affected by different psychiatric disorders; the methods of suicide attempts were not controlled in all studies. Differences may exist between participants and those that did not accept to participate in the studies. Furthermore, some of these studies may lack a control group or, alternatively, controls were not matched for age and other clinically relevant information. In addition, psychometric instruments evaluating suicide risk were not used in all studies. 

Moreover, many of the observed abnormalities in neuropeptides concentrations were found in subjects with suicidal behavior who were also depressed. It is possible that the association between neuropeptides and suicidality may be mediated by the existence of psychiatric conditions such as major affective disorders. Generally, the variability in the concentrations of neuropeptide markers is too great to draw any definitive conclusion in those who were depressed and also displayed suicidality. 

In light of recent advances in understanding the regulation of genes encoding neuropeptides, neuropeptide receptors have emerged as attractive targets for the treatment of many psychiatric disorders. To date, many molecules have been synthesized but none has been introduced into the market due to the failure of most clinical trials aimed to show their efficacy. Griebel and Holsboer [146] stated in their review that although no strong mechanistic hypothesis supports the active role of the neuropeptide system in psychiatric conditions, medications targeting neuropeptide receptors have not completely failed to demonstrate their efficacy. They analyzed preclinical and clinical evidence on nonpeptidergic ligands for neuropeptide receptors in psychiatric disorders mainly focusing on tachykinins, CRF, vasopressin, and neurotensin. Overall, the authors suggested the clinical potential of neuropeptide receptor antagonists as candidate medications that may help to reduce the toll of major affective disorders and suicide but reported the importance to stratify patients according to the different subtypes and illness severity. Objectifiable laboratory tests should be available to go beyond the issue of heterogeneity of patients recruited in the different studies. Moreover, the authors recommended the careful knowledge of DNA sequence variations of neuropeptides and they highlighted the importance to ensure that abnormalities in neuropeptide signalling represent one of the most relevant pathogenetic factors in major depression.

Biomarkers and genetic tests are suggested to identify clinical conditions that may be related to a specific neuropeptidergic mechanism. The specificity of the mechanism of action of the different neuropeptides should also be adequately considered. An important research question that needs to be addressed in future studies is how and when personalizing treatments using drugs targeting neuropeptide receptors are possible. This implies the knowledge of whether subjects could preferably respond to a medication or another, which may be the right combination of medications, and what is the real benefit for some subgroups of patients.

The possibility to select medications targeting neuropeptide receptors according to findings derived from animal studies should be carefully considered using models evaluating different aspects of the disorders. 

A detailed characterization of patients based on objectifiable measures, genetic tests, and reliable biomarkers is undoubtedly requested.

## 5. Conclusion

According to most of the studies included in the present review, neuropeptides may play a key role in the pathophysiology of suicidal behavior. In particular, one mechanism by which neuropeptide alterations may promote suicidal symptoms is via changes in monoaminergic systems rendering neuropeptides as novel, intriguing mediators of stress-related and affective conditions as well as suicidality. 

However, despite the recent advances in this research field, it is unlikely that neuropeptides represent definitive and reliable markers of suicidality. Further additional studies will elucidate the pathophysiological mechanisms of the different neuropeptides pathways underlying suicidal behavior.

## Figures and Tables

**Table 1:** Neuropeptides that may be relevant in major affective disorders and suicidal behavior.

Corticotropin-releasing factor	CRF is a 41-amino-acid neuropeptide that binds the G-protein-coupled receptors CRF1 and CRF2. The association between CRF/CRF1 system and stress-related disorders is well known [23, 37–39]. CRF is mainly implicated in the regulation of the HPA axis, the abnormalities (increased cerebral spinal fluid CRF, hyperreactivity of the HPA axis, basal hypercortisolemia, increased release of adrenocorticotropin hormone (ACTH) by CRF in subjects treated with dexamethasone, and reduced corticosteroid receptor mediated feedback inhibition of the HPA axis) of which have been frequently associated with psychiatric conditions [40]. It has been demonstrated that antidepressant treatment may restore HPA-axis abnormalities [41], particularly in the paraventricular nucleus of the hypothalamus [42], enhancing the corticosteroid receptor (CR) hypothesis based on the impaired CR function in the pathophysiology of major depression [43]. The hyperactivity and dysregulation of CRF system may trigger and/or involve the maintenance of these alterations as chronic stress or depression have been associated with increased limbic levels of CRF [44–46]. The hyperactivity of CRF system has been associated with reduced hypophyseal CRF binding in depressed patients [47] and reduced corticosteroid feedback inhibition [48]. Moreover, CRF is also implicated in emotional regulation, learning and memory, and autonomic and monoaminergic modulation which are involved in the pathogenesis of major depression [37, 49]. Relevantly, CRF antagonism modulates alterations in many other neuropeptides and hormones systems [30]. Overall, there is a large body of evidence suggesting the involvement of CRF in the pathophysiology of major affective disorders [50–53] but not all studies replicated these findings [54].

Arginine vasopressin	AVP is a nine-amino-acid neuropeptide released by the magnocellular terminals into the posterior pituitary mediating the resorption of water in the kidney. AVP interacts with CRF in the parvocellular neurons with the final aim to modulate HPA-axis activity. AVP transmission is also involved in different functions such as learning and memory, aggression, and sociality [55]. AVP binds V1a, V1b, and V3 G protein-coupled receptors which are associated with vasoconstriction, ACTH release, and antidiuresis. Parvocellular AVP and CRF are known to regulate the HPA axis. Several findings including those derived by postmortem analyses suggested that AVP was increased in either the brain or plasma of depressed patients [56, 57]. Stressful stimuli lead to persistent increase of AVP in CRF neurons without inducing CRF changes [58]. ACTH release and responsiveness induced by CRF effects are enhanced by AVP [59]. Dinan and Scott [60] suggested that prolonged AVP transmission is a critical mechanism in determining HPA axis hyperactivity in depressed patients. There is evidence that genetic variation of AVP as well as its genetic inactivation in animals [61, 62] was related to childhood-onset mood disorders and anxiety-related behaviors. Furthermore, AVP antagonists have demonstrated efficacy in anxious and depressive human conditions showing anxiolytic and antidepressant properties [63, 64]. Overall, there are consistent evidence suggesting an active involvement of AVP in the pathogenesis of major affective disorders and recently several AVP antagonists showed promising results as possible antidepressant and anxiolytic medications [65].

Oxytocin	Oxytocin is a nonapeptide binding to a single G-protein coupled receptor and mainly implicated in behaviors such as childbirth, lactation and sexual behaviors, social memory, and cognition. Oxytocin is synthesized in the paraventricular and supraoptic nuclei of the hypothalamus, transported to the posterior pituitary and released to amygdala, hypothalamus, hippocampus, and nucleus accumbens. According to animal studies, the infusion of oxytocin stimulates maternal behavior and the administration of antagonists inhibits this behavior [66]. Social memory has been demonstrated to be influenced by the oxytocin system in laboratory animals and humans. Oxytocin antagonist may inhibit social recognition in rats that was enhanced by the infusion of an oxytocin agonist into the lateral ventricles [67]. Findings from animal studies suggested that oxytocin represents a critical modulating neuropeptide in the regulation of social interaction. Recently, it has been demonstrated that recognition memory for faces but not nonsocial stimuli has been enhanced in humans by intranasal administration of oxytocin [68]. Furthermore, oxytocin both in rodents [69] and human subjects [70] may influence the stress response modulating the HPA axis and reducing the release of stress hormone. The anxiolytic activity of oxytocin and oxytocin agonists that may be inhibited by oxytocin antagonists has been also reported in several preclinical tests [71, 72]. Intranasal oxytocin has been suggested to reduce the activation of the amygdala in response to fearful or threatening scenes in human individuals [73]. Reduced plasma levels of oxytocin have been found in patients with MDD [74] and in the cerebrospinal fluid (CSF) of schizophrenic patients [75]. Oxytocin agonists have also been reported to be effective in preclinical tests of antidepressant activity such as the tail suspension test [72]. However, CSF oxytocin has also been suggested as an important modulator of suicidal intent and interpersonal violence. Jokinen et al. [76] found that 28 medication-free suicide attempters had lower CSF oxytocin levels compared to 19 healthy controls. Among the suicide attempters, CSF oxytocin levels correlated significantly and negatively with the planning subscale of the Beck Suicide Intent Scale (SIS). After regression analyses, suicide intent was a significant predictor of CSF oxytocin corrected for age and gender whereas lifetime violent behaviour showed a trend to be a predictor of CSF oxytocin. Moreover, oxytocin agonists have been reported to restore prepulse inhibition that is disrupted by N-methyl-D-aspartate (NMDA) antagonists or dopamine agonists in rats [72, 77]. Intranasal administration of oxytocin was also reported to be useful as an adjunctive treatment in patients with schizophrenia who were treated with antipsychotic medications [78]. Finally, reduced oxytocin plasma levels have been demonstrated in autistic children [79] and several single nucleotide polymorphisms in the oxytocin system have been associated with autism [80]. Speech comprehension and social recognition [81] as well as repetitive behaviors [82] may be improved in autistic patients with administration of oxytocin.

**Table 2:** Most relevant studies about the association between neuropeptides and suicide.

Author(s), year	Sample characteristics	Study design and psychometric instruments	Type of neuropeptide which has been investigated	Main findings	Limitations	Conclusions
Lindqvist et al., 2011 [83]	124 patients who attempted suicide (63 men, 61 women; mean age 38 ± 14), of which 35 with MDD, 13 with depression not otherwise specified (NOS), 28 with adjustment disorder, 17 with dysthymia, and 31 with other DSM-IV diagnoses.	Patients were evaluated with the Montgomery-Asberg Depression Rating Scale (MADRS), the SIS, and the Suicide Assessment Scale (SUAS). Suicide attempts were divided into violent and nonviolent acts. Principal component analysis of CSF biomarkers were carried out.	Lumbar punctures were performed and samples were stored in aliquots and immediately frozen in −80°C. The following substances were quantified in CSF: eotaxin, eotaxin-3, interferon-gamma-inducible protein-10, interleukin-(IL-) 1*β*, IL-6, IL-8, macrophage-derived chemokine, macrophage inflammatory protein-1*β*, matrix metalloprotease, MMP-3, MMP-9, monocyte chemotactic protein-1, monocyte chemotactic protein-4, orexin, thymus, activation-regulated chemokine, tumor necrosis factor-*α*, kynurenic acid, 5-hydroxyindoleacetic acid (5-HIAA), homovanillic acid (HVA), and 3-methoxy-4-hydroxyphenylglycol.	Factor 4 characterized by positive loadings of monoamine metabolites 5-HIAA and HVA, the proinflammatory cytokine IL-6, and negative loading on the HPA-axis-associated neuropeptide orexin was associated with violent suicide method, risk for suicide completion, and less impulsivity.	The study did not include a control group. A small subset of the suicide attempters had somatic diagnoses. Whether storage time might have influenced the biomarkers is unknown.	Analyzing clusters of biomarkers in the suicidal patients may improve the clinical assessment of future suicide risk.

Stanley et al., 2010 [84]	29 psychiatric patients with a history of one or more suicide attempts with a mean of 3.0 attempts (SD = 2.4).	Patients were assessed using the Hamilton Rating Scale for Depression (HRSD), Beck Depression Inventory (BDI), Brief Psychiatric Rating Scale (BPRS), Clinical Global Impressions Scale (CGI), and Buss-Durkee Hostility Inventory. The sample was divided according to the presence or absence of a history of repeated nonsuicidal self-injury (NSSI)	Cerebrospinal fluid samples were collected via lumbar puncture, and CSF *β*-endorphin, met-enkephalin, dynorphin, serotonin metabolite, 5-HIAA, and dopamine metabolite, HVA were measured.	Patients in the NSSI group had lower CSF *β*-endorphin levels and met-enkephalin when compared with a matched group of patients without NSSI but no difference in dynorphin levels was found between the two groups. Also, metabolite levels did not differ in relation to NSSI.	Plasma neuropeptide levels may not accurately reflect central levels. Whether results are due in part to the stress response of individuals with NSSI is unknown.	Patients with NSSI have abnormalities in the brain opioid system suggesting the possibility of disordered pain or reward circuitry. *β*-endorphin and met-enkephalin, opioids acting upon receptors involved in mediating stress-induced and physical pain analgesia, are all involved in NSSI.

Brundin et al., 2009 [85]	4 MDD males and 6 MDD females of 41 ± 9 years old (SD ± 1) during the year after a suicide attempt.	Patients were evaluated using the SUAS and Comprehensive Psychopathological Rating Scale (CPRS) at baseline, after 6 and 12 months.	Lumbar punctures were performed and samples stored in −80°C. CSF-orexin was analyzed.	CSF-orexin increased significantly between the suicide attempt and the first follow-up, from 161.3 ± 19.0 pg/mL to 182.6 ± 30.0 pg/mL (*P* = 0.021). Orexin was higher in all patients but one (the mean pairwise increase was 28.5 ± 9.0 pg/mL). At the second follow-up after one year, mean CSF-orexin was still significantly higher than at the time of the suicide attempt (183.3 ± 19.0 pg/mL).	The small number of subjects limits the generalization of findings. Also, the number of patients in the group receiving antidepressive medications was too small for any conclusions to be drawn concerning its effect on CSF-orexin.	CSF-orexin increased significantly during the first year after a suicide attempt and was associated with an improvement (reduction) of the SUAS scores.

Pitchot et al., 2008 [86]	28 MDD (19 men and 9 women) inpatients were divided into 13 suicide attempters and 15 nonattempters	No psychometric instruments were used.	Neurophysin blood level which is considered a better method to investigate neuropituitary function was used. Basal plasma levels of AVP neurophysins extracted at 8:00 am and postdexamethasone suppression test (DST) cortisol levels were measured.	There was no correlation between HRSD scores and AVP-neurophysins levels or post-DST cortisol concentrations. Any significant correlation between AVP-neurophysins and post-DST cortisol levels was observed. AVP-neurophysins did not differ between DST suppressors and nonsuppressors.	AVP in plasma reflects neuropituitary AVP release and not AVP activity in the magnocellular portion of the paraventricular nucleus. Also, the study was limited by the weak statistical power.	Results fail to support a possible role of AVP-neurophysins in the control of suicidal behavior.

Brundin et al., 2007 [87]	66 patients (with a mean age of 39 ± 14.3), 32 with MDD, 11 dysthymia and 23 an adjustment disorder after a suicide attempt.	Patients were evaluated using the CPRS.	Lumbar puncture performed in the morning between 08.00 and 09.00 am after a night of fasting and bed rest and CSF orexin-A levels were measured.	The orexin level was significantly lower in the group of patients with MDD compared to patients with adjustment disorder and dysthymia. Orexin correlated significantly with CSF-levels of somatostatin, delta sleep inducing peptide-like immunoreactivity (DSIP-LI), and CRF.	The study lacks a group of healthy controls. The range of orexin levels in normal subjects is large. Also, most of the patients who attempted suicide by intoxication used high amounts of benzodiazepines and several patients also received sedatives during the wash-out period.	Orexin neurotransmission may be a relevant therapeutic target in the pathogenesis of MDD

Brundin et al., 2007 [88]	101 suicide attempters (51 male and 50 female with a mean age of 39 ± 15), 31 with MDD, 23 with adjustment disorder, 11 with dysthymia, 14 with depressive syndrome NOS, and 22 with other diagnoses.	Patients were evaluated using the CPRS.	Lumbar puncture was performed to measure CSF orexin-A.	Significant negative correlations between lassitude and CSF-orexin levels, slowness of movement and CSF-orexin levels and rating of global illness and CSF-orexin levels.	It cannot be ruled out that some other symptoms not assessed in this study may be associated with low CSF-orexin levels.	Low orexin levels were associated with specific psychiatric symptoms (specifically, inertia and decreased motor activity).

Austin et al., 2003 [89]	11 subjects who died by suicide and having a diagnosis of depression were matched with control subjects.	No psychometric instruments were used.	Postmortem study aimed to evaluate corticotropin-releasing hormone (CRH) levels.	The mean level of CRH-IR in depressed subjects was significantly increased by 30% in the LC, by 45% in the caudal nucleus of the DR, and by 39% in the median raphe relative to controls. The mean level of CRH-IR was not significantly different in the medial parabrachial nucleus and dorsal tegmental nucleus of depressed subjects than controls. A significant positive correlation was found between the magnitude of the increase in CRH-IR levels in the LC and the age at onset of depression.	The study was conducted only in male subjects. The small sample size limits the generalization of findings.	CRH-IR levels were increased in the LC, caudal nucleus of the DR, and median raphe of depressed suicide men compared to controls. CRH neurotransmission is increased in the extrahypothalamic brain regions of depressed subjects.

Merali et al., 2006 [90]	Brain samples were obtained from suicides (24 men of 48.13 ± 2.53 years; 6 women of 49.17 ± 6.77 years) of which 27 with MDD (recurrent episodes), 1 with bipolar disorder—depressed phase, and 2 with psychotic MDD and controls (23 men of 51.82 ± 1.96 years and 14 women of 57.36 ± 4.08 years).	No psychometric instruments were used.	Brains were obtained 1–6 hours postmortem to measure CRH, AVP, gastrin-releasing peptide (GRP), and neuromedin B (NMB) levels.	Levels of CRH-ir among suicides were increased in the LC, frontopolar, dorsolateral prefrontal, and ventromedial prefrontal cortices but were reduced at the dorsovagal complex. The concentration of AVP-ir was increased at the paraventricular hypothalamic nucleus, LC, and dorsolateral prefrontal cortex and reduced at the dorsovagal complex (DVC).	The considered number of suicides and controls for each brain region was relatively small (there was insufficient power to determine the contribution of gender to the observed peptide differences between suicides and controls). Whether age differences between suicides and controls may account for the observed outcomes is unknown.	Suicide was associated with site-specific alterations in the endogenous levels of CRH, AVP, GRP, and NMB. However, the exact clinical significance of these peptidergic changes in the suicide brain is unclear.

Brunner et al., 2002 [91]	19 inpatient suicide attempters (12 female, 7 male) aged 38 ± 3 years. 6 patients had MDD (single episode), 3 MDD (recurrent episode), 2 had bipolar disorder, 7 an adjustment disorder, and 1 dysthymic disorder.	Depressive symptoms were assessed using HDRS_21_.	Lumbar puncture was performed to measure CSF and plasma AVP, CSF, and plasma HVA and plasma N^+^ and K^+^.	There were no differences between all 19 depressed subjects and 9 controls with respect to all biochemical parameters measured in CSF plasma. Suicide attempters did not differ from nonattempters. In depressed patients, plasma AVP correlated positively with cortisol. There was no relationship between CSF AVP and monoamine metabolites in CSF.	The small sample size and the heterogeneous diagnoses in the patient population did not allow to rule out a possible pathogenic role of central AVP in suicidal behavior.	No differences in the CSF AVP concentrations between depressed suicide attempters, depressed nonsuicidal patients, and neurological control subjects were reported.

Caberlotto and Hurd, 2001 [92]	44 subjects died by suicide with a mean age of 44.1 (6 females and 9 males with MDD, 6 females and 9 males with bipolar disorder, and 5 subjects with other diagnoses) compared to 15 controls (9 males, 6 females) with a mean age of 48.1 years.	No psychometric instruments were used.	Postmortem study aiming to measure Y1 and Y2 receptor mRNA expression levels.	No significant alterations in Y1 or Y2 mRNA expression levels were observed between the groups. The Y2 mRNA expression was elevated in layer IV in subjects with suicide as a cause of death. For the Y1 mRNA expression, there was a negative correlation with increasing subject age in the prefrontal cortex.	Information regarding detailed toxicology and the history of other antipsychotics or antidepressant medications was not available. Several post-mortem parameters may affect the stability of mRNAs and proteins in the human brain. The Y2 mRNA expression was significantly influenced by post-mortem delay.	There are indices of an impairment of the prefrontal cortical NPY receptor mRNA expression in suicide, but this might not relate to the pathophysiology of mood disorders.

Inder et al., 1997 [93]	45 MDD patients (27 females and 18 males) aged 32.5 ± 1.5 years (34 (75.6%) of the patients had suicidal ideation and 7 had attempted suicide within the current month) compared with 11 healthy controls (4 males and 7 females) aged 32.5 ± 3.0 years.	Depressive symptoms were assessed using HDRS_17_.	Levels of cortisol, ACTH, AVP, and CRH were measured.	There was a significant correlation between ACTH and cortisol, AVP and ACTH, and AVP and cortisol. No significant correlation was observed between plasma CRH and either ACTH or cortisol. There was no relationship between severity of depression as measured on HDRS score and any of the hormone parameters measured. Also, there was no difference in mean cortisol between depressed subjects and controls. Plasma AVP was significantly higher in patients who had attempted suicide.	Although plasma AVP levels were higher in those subjects who had attempted suicide compared to those who did not, differences in ACTH and cortisol levels were not significant. The sample is too small to generalize findings.	There is a significant positive correlation between plasma AVP, ACTH, and cortisol levels in depressed subjects. Also, plasma AVP levels are increased in those subjects who had attempted suicide.

Olsson et al., 2004 [94]	13 patients who attempted suicide diagnosed with MDD according to the DSM-III-R criteria.	Patients were assessed using MADRS and the Brief Anxiety Scale (BSA), examined at pretreatment, and followed by every 3 or 4 months.	Lumbar punctures were performed to examine CSF NPY and SP levels.	Antidepressant treatment seemed to affect the levels of CSF NPY and SP, which decreased significantly between the second and last lumbar puncture. At pretreatment, BSA scores were significantly and negatively correlated with CSF SP and tended to be negatively correlated with CSF NPY. There were also significant positive correlations between CSF NPY and SP in the whole group, possibly reflecting an interrelationship between these neuropeptides.	The small number of patients might have influenced the results. Also, patients were included because of an attempted suicide and not because of a specific diagnosis. The drugs that some of the patients ingested in order to intoxicate themselves may be another confounding factor.	Long-term antidepressant treatment had no specific effect on SP in patients who attempted suicide. It is possible that NPY was affected by long-term antidepressant treatment.

Westrin et al., 2001 [95]	16 patients (11 patients with previous suicide attempts). 4 patients had MDD, 5 dysthymia, and 7 other diagnoses.	Drug-free patients were evaluated with the SUAS and MADRS at baseline and after a median of 7 (5 to 9) months.	Lumbar punctures were performed to measure CSF-CRH and CSF somatostatin alterations.	At follow-up, MADRS and SUAS scores were significantly decreased (*P* < 0.05), whereas CSF—somatostatin was significantly increased (*P* = 0.013) and CSF-CRH had not changed significantly at follow-up.	The substances ingested might have caused the initially low CRH and somatostatin levels. The study did not show whether somatostatin changes are specifically related to either suicidality or depression.	CSF somatostatin but not CSF-CRH seems to increase with clinical improvement in suicidal patients, independently of psychiatric diagnoses.

Westrin et al., 1999 [33]	36 suicide attempters (mean age 37 ± 14), of which 12 with MDD, 12 depressive disorder NOS, 2 dysthymia, and 12 adjustment disorder. 11 patients had made one or more previous suicide attempts. Patients were compared with 36 controls (mean age 37 ± 14).	Patients were evaluated using MADRS and SUAS.	Serum and plasma cortisol, CRH, NPY, and beta-endorphin (beta-END), were measured.	When compared with healthy controls, cortisol was high (*P* < 0.001) and CRH and NPY were low (*P* < 0.001) in recent suicide attempters. Patients who had repeatedly attempted suicide had the lowest NPY. A correlation between NPY and cortisol (*P* < 0.05) was found in suicidal patients with depression NOS, whereas beta-END correlated with cortisol (*P* < 0.01) in suicidal MDD patients. A postdexamethasone decrease of NPY was observed only in controls.	Whether the observed alterations depend on the underlying depression or stress associated with a suicide attempt is unknown. The small sample size did not allow the generalization of findings.	There may be alterations in CRH and NPY plasma levels in patients with a mood disorder who were recent suicide attempters.

Westrin et al., 1998 [96]	34 patients (mean age 37 ± 14), of which 12 had MDD, and 22 other major affective disorders were compared with 34 controls (mean age 37 ± 15).	Patients were evaluated using MADRS and SUAS.	DSIP-LI levels were measured.	Significantly elevated DSIP-LI levels in MDD patients and a significant correlation between predexamethasone cortisol and predexamethasone DSIP-LI levels in healthy controls were found. Postdexamethasone DSIP-LI levels increased in subjects with low predexamethasone DSIP-LI levels whereas they decreased in subjects with high predexamethasone DSIP-LI levels.	A nonsuicidal control group was not included. The small sample size did not allow to generalize findings.	Suicidal MDD patients and patients with previous suicide attempts had increased plasma DSIP levels.

Westrin et al., 1998 [97]	A total of 38 suicide attempters (mean age of 38 ± 14) of which 11 with MDD, 12 with adjustment disorder, 10 with depression NOS were compared with 38 controls (mean age of 38 ± 14). 18 had a personality disorder.	Patients were assessed using Karolinska Scales of Personality, the Eysenck Personality Questionnaire (EPQ), Impulsiveness-Venturesomeness-Empathy (IVE) Inventory (EPQI) and the Marke-Nyman Temperament (MNT).	Serum and plasma samples for cortisol, DSIP-LI, CRH-LI, and NPY-LI were measured.	NPY correlated positively with psychasthenia, irritability, and stability and negatively with validity in patients, but negatively with muscular tension, psychasthenia, verbal aggression, and irritability in controls. DSIP correlated positively with impulsiveness (EPQI) in controls. CRH correlated negatively with the temperament dimension of lie in controls. Cortisol correlated positively with validity, extraversion and verbal aggression and negatively with inhibition of aggression in controls.	Nonsuicidal patients were not included in the analysis. The small sample size did not allow to generalize findings.	Different correlational patterns in patients and controls are probably due to plasma peptides or cortisol as well as some temperament dimensions being state rather than trait related.

Roy, 1993 [98]	Depressed patients who had attempted and repeated suicide after the 5-year follow-up period.	A 5-year follow-up was performed	CSF concentrations of NPY, somatostatin, diazepam-binding inhibitor, gamma-aminobutyric acid (GABA), or CRH were analyzed.	There were no significant differences between depressed patients who did or did not repeat suicide during the follow-up or who had never attempted for CSF concentrations of the NPY, somatostatin, diazepam-binding inhibitor, GABA, or CRH.	A non-suicidal control group was not included.	NPY, somatostatin, diazepam-binding inhibitor, GABA, or CRH are not major determinants of suicidal behavior or its repetition in depression

Widdowson et al., 1992 [34]	Suicide victims and individuals dying a sudden natural or accidental death (controls).	No psychometric instruments were used.	Post-mortem study in which NPY-concentrations in frontal (BA 10) and temporal cortex (BA 22), caudate nucleus, and cerebellum were analyzed.	NPY levels were significantly lower in postmortem frontal cortex (−14%) and caudate nucleus (−27%) from suicide victims compared with age-matched controls. Suicides with a history of depression displayed more robust reductions in NPY immunoreactivity in frontal cortex and caudate nucleus, as did 4 subjects who died from natural causes and were also described as having a possible history of depression.	Other post-mortem parameters that were not investigated may have affected NPY concentrations in the human brain. Subjects were not evaluated with psychometric instruments.	An NPY deficit in the brain leading to region-specific reductions in peptide concentrations in subjects who have a history of depression was found.

Ordway et al., 1995 [99]	Suicide victims having MDD established by psychiatric autopsy compared to controls (who had died by natural or accidental death).	No psychometric instruments were used.	Post-mortem study in which NPY concentrations in frontal cortex were analyzed.	No significant differences in NPY concentrations were observed between control subjects and suicide victims with MDD or alcohol dependence.	Several subjects with MDD had a comorbid diagnosis of alcoholism.	The possible role of NPY in MDD was not confirmed.

Träskman-Bendz et al., 1992 [100]	44 suicide attempters diagnosed with MDD.	Patients were evaluated using MADRS.	Lumbar punctures were performed to measure the following HPA-axis related peptides: CRH, somatostatin, DSIP, NPY, beta-END, and AVP; serotonin metabolite 5-HIAA in CSF and postdexamethasone plasma cortisol were also measured.	Strong correlations between CRH and the peptides somatostatin and beta-END, with the latter also correlated positively with somatostatin. There were no gender differences. Patients with MDD had significantly lower somatostatin, CRH, and DSIP than other patients. Both somatostatin and beta-END correlated negatively with postdexamethasone plasma cortisol in all patients. No significant relationships between neuropeptides and CSF 5-HIAA were found. Patients who had made previous suicide attempts had significantly lower CRH than those who had not.	The study was cross-sectional in nature. Other neuropeptides which were not investigated may affect the HPA regulation.	There was no indication of specific neuropeptide patterns in those MDD patients who later completed suicide.

Scarone et al., 1990 [101]	7 suicides and 7 dying a sudden natural death (controls).	No psychometric instruments were used.	Post-mortem study in which beta-endorphin levels were analyzed.	Reduced beta-endorphin levels in the left temporal cortex, left frontal cortex, and left caudate nucleus of suicides compared to controls were found. An asymmetrical concentration of beta-endorphin in suicides (left less than right) in frontal cortex and caudate nucleus was also found.	The small sample size did not allow to generalize findings.	Suicidal behaviour might be related to the lateralized mechanisms of mood control.

Maes et al., 1989 [102]	17 suicidal and 17 non-suicidal female MDD patients matched for age and severity of illness. Subjects were divided in to those with suicidal ideation and those without.	No psychometric instruments were used.	Thyroid-stimulating hormone (TSH), free thyroxin (FT4), pre- and postdexamethasone cortisol, ACTH levels, the circulating concentrations of total L-tryptophan (L-TRP), and the ratio between L-TRP and competing amino acids (CAA) were measured	No significant differences in any of the examined biological data between patients with suicidal ideation and those without.	The study was cross-sectional in nature. Subjects were not evaluated with psychometric instruments.	The possible role of TSH, FT4, ACTH levels, and L-TRP was not confirmed in suicidal patients.
